# Short-Term and Intermediate Efficacy of Tacrolimus for Active Ulcerative Colitis: A Single-Center Retrospective Analysis in Japan

**DOI:** 10.7759/cureus.73552

**Published:** 2024-11-12

**Authors:** Satoshi Masuyama, Mimari Kanazawa, Keiichi Tominaga, Takanao Tanaka, Shunsuke Kojimahara, Shoko Watanabe, Akira Yamamiya, Takeshi Sugaya, Yasuo Haruyama, Atsushi Irisawa

**Affiliations:** 1 Department of Gastroenterology, Dokkyo Medical University School of Medicine, Tochigi, JPN; 2 Integrated Research Faculty for Advanced Medical Sciences, Dokkyo Medical University School of Medicine, Tochigi, JPN

**Keywords:** clinical remission, remission induction therapy, short-term and intermediate efficacy, tacrolimus, ulcerative colitis

## Abstract

Background and aim

Tacrolimus (tac) is used for induction therapy in refractory and severe ulcerative colitis (UC) cases. The aim of this study was to identify the factors contributing to the induction of remission and to assess the endoscopic or histologic improvement rates following induction of remission by tac.

Methods

This study examined data from 67 UC patients treated with tac for induction of remission out of 515 patients attending Dokkyo Medical University Hospital. The primary endpoint of the study was the analysis of factors contributing to successful induction of remission treatment with tac. The secondary endpoints were the corticosteroid-free remission rate at 52 weeks after tac induction and the endoscopic and histologic improvement rates following induction of remission.

Results

Analysis of factors contributing to successful induction of remission by tac showed the Lichtiger index at the beginning of remission induction therapy was 9.5 ± 2.5 for the successful remission group and 11.5 ± 2.4 for the unsuccessful remission group (p = 0.002). The proportions of patients who had used immunomodulators were 13/45 (28.9%) for the successful remission group and 14/22 (63.6%) for the unsuccessful remission group (p = 0.006). The proportions of patients who had used anti-tumor necrosis factor (TNF)α biologics were 4/45 (8.9%) for the successful remission group and 8/22 (36.4%) for the unsuccessful remission group (p = 0.006).

Conclusion

Patients with UC who are potential candidates for intensification of remission maintenance therapy are good candidates for induction of remission with tac. Moreover, improvement in endoscopic inflammation might be a predictive marker of response to remission induction therapy with tac.

## Introduction

Ulcerative colitis (UC), a chronic inflammatory bowel disease (IBD) of unknown etiology [1], is characterized by repeated relapses and remissions. It presents symptoms such as abdominal pain, bloody stool, diarrhea, fever, and weight loss. Because no fundamental therapeutic strategy has been established, the goal of treatment is generally to maintain the remission phase for a long period after remission induction therapy [1,2]. Nevertheless, some patients are resistant to or dependent on corticosteroids (CS), which are first-line remission-inducing agents [2,3]. Tacrolimus (tac) and cyclosporine (CyA) are calcineurin inhibitors used to treat refractory and severe UC cases. In Japan, CyA has not been approved by the Ministry of Health, Labour, and Welfare for UC in clinical practice, but tac is often used [4,5]. Tac is an immunosuppressant agent identified from a metabolic product of Streptomyces tsukubaensis. It inhibits the dephosphorylation of calcineurin and blocks the transcription of genes such as interleukin (IL)-2, thereby exerting its medicinal effects as an immunosuppressant. Moreover, tac exhibits an effect on macrophages, inhibiting the release of IL-12 and tumor necrosis factor (TNF) α from macrophages after lipopolysaccharide stimulation in a concentration-dependent manner [6,7]. Randomized trials demonstrating the efficacy of tac in remission induction therapy for active UC have shown significantly higher rates of short-term remission, symptom improvement, and endoscopic improvement versus placebo [8]. Although several reports have described outcomes of remission induction therapy with tac for patients with UC, few reports have studied factors contributing to remission induction or endoscopic or histologic improvement after remission induction therapy with tac.

The study aims to assess specific factors that influence successful remission induction and improvement in endoscopic and histologic findings after tac induction in patients with UC. In addition, the usefulness and positioning of tac are discussed.

## Materials and methods

Patient characteristics and UC treatment strategies

**Figure 1:**
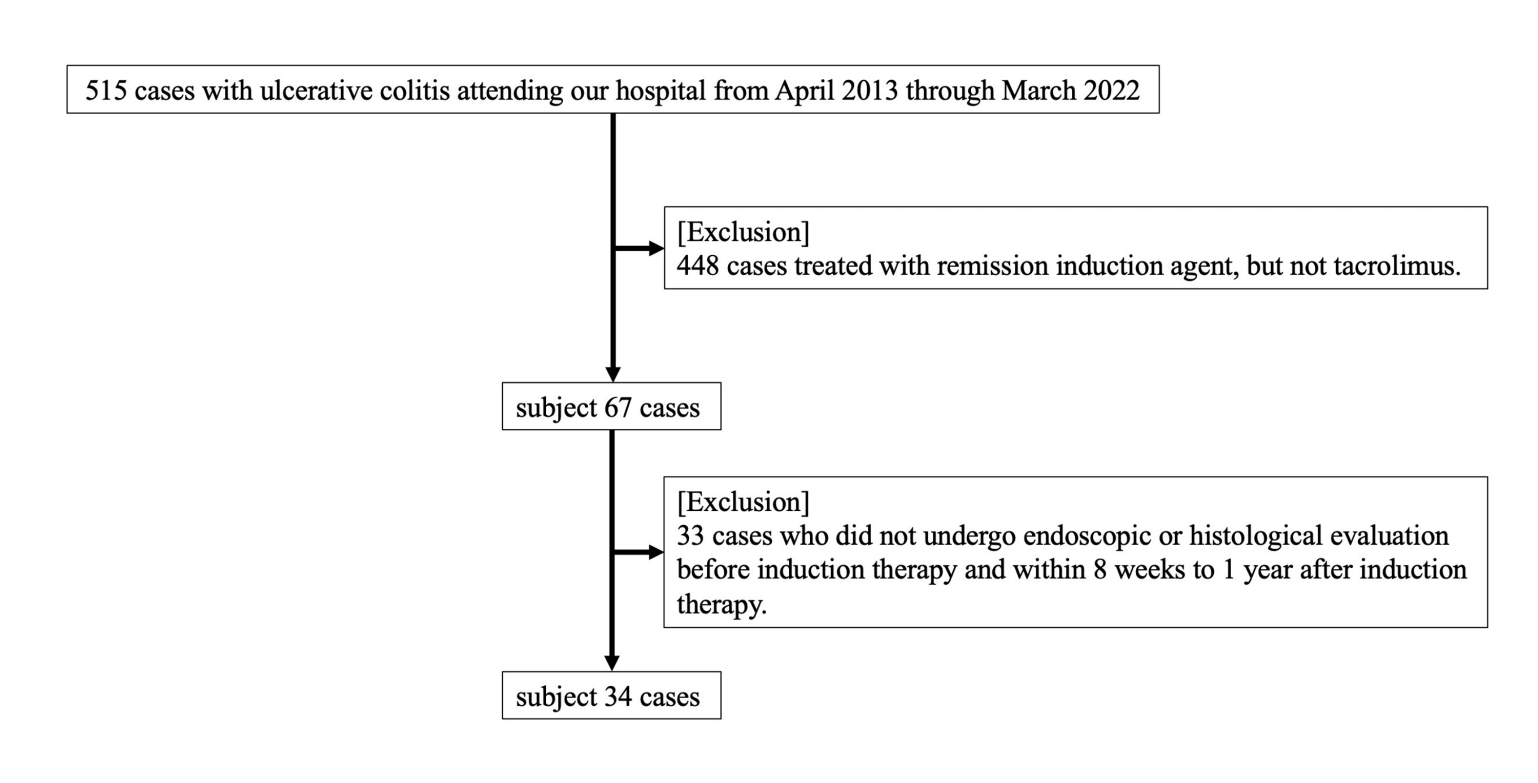
Patient inclusion and exclusion criteria.

This study examined data from 67 UC patients aged 12 to 70 years who were treated with tac for induction of remission out of 515 patients attending Dokkyo Medical University Hospital from April 2013 to March 2022. At our institution, remission induction therapy for active UC is administered according to the Japanese evidence-based clinical practice guidelines for IBD [4,5]. Tac is used as an induction remission agent in refractory cases, such as those that are steroid-resistant and steroid-dependent. The analysis of endoscopic and histologic improvement rates included 34 patients for whom endoscopic and histologic evaluations were possible before and within eight weeks to one year after induction remission therapy with tac (Figure 1). Although granulocyte and monocyte adsorption apheresis (GMA) can independently reduce inflammation, all GMA combination cases were aimed to improve the symptoms by adding GMA to the first remission induction agent (mostly PSL). In this study, we included unsuccessful cases of first remission induction therapy. In other words, all cases using GMA were unsuccessful in inducing remission with GMA. Although some patients were treated with GMA in the early stages of tac induction, none were treated with GMA at the end of the remission induction. Medical information was obtained from electronic medical records, including age, gender, disease type, clinical course, clinical severity (Lichtiger index), history of immunomodulators (IM) and molecular-targeted agents, C-reactive protein (CRP) and albumin levels before starting treatment, initial tac trough value, time to reach the therapeutic range, endoscopic and histological improvements, steroid-free remission, 5-aminosalicylic acid (5-ASA) intolerance, and additional therapy after induction of remission.

Evaluation of ulcerative colitis severity

Clinical activity was assessed using the Lichtiger index [9]. A Lichtiger index cut-off of 11 or higher was considered severe. A cut-off of 3 or lower was inferred as remission. Clinical improvement was defined as a reduction of 50% or more from the baseline score.

Treatment regimens and agents

The therapeutic agents used for this study were approved for use in Japan. Tac (Prograf®; Astellas Pharma Inc.) was administered orally using capsules. The initial dose of tac was 0.05 mg/kg twice daily. The target trough concentration of tac was 10-15 ng/mL in the remission-induction phase and 5-10 ng/mL in the remission-maintenance phase. Blood tac concentrations were measured twice a week for inpatients and twice a month for outpatients during the remission-induction phase. Although tac therapy over three months has not been approved in Japan, for this study, tac treatment was either discontinued after 3 months or continued for longer periods at the discretion of the primary physician. For this study, we used 5-ASA as a time-dependent ASA (2000-4000 mg/day, Pentasa®; Kyorin Pharmaceutical Co. Ltd.), a pH-dependent ASA (2400-3600 mg/day, Asacol®; Zeria Pharmaceutical Co. Ltd.), a pH-responsive ASA (2400-4800 mg/day, Lialda®; Mochida Pharmaceutical Co. Ltd.), and salazosulfapyridine (2000-4000 mg/day, Salazopyrin®; Pfizer Japan Inc.). No generic agent was used.

Definition of refractory cases and corticosteroid-free remission

Refractory UC, as described in the Japanese evidence-based clinical practice guidelines for IBD, has been defined as (1) CS resistance (ineffectiveness of CS at 1-1.5 mg/kg/day for 1-2 weeks) or CS dependence (relapse during CS tapering) despite strict CS therapy, or (2) frequent relapse or chronic continuous cases while under strict medical therapy except for CS [4,5]. For this study, CS-free remission was defined as having reached clinical remission in the absence of CS medication. All patients were CS-free at the end of remission induction therapy.

Endoscopic and histological evaluation

Endoscopic evaluation was performed by experienced endoscopists from the Japan Gastroenterological Endoscopy Society, who are particularly skilled in IBD treatment. The Mayo Endoscopic Subscore (MES) was used to assess endoscopic activity: MES 0 (no friability, granularity, or intact vascular pattern) was defined as normal; MES 1 (mild erythema or decreased vascular pattern) was defined as remitted mucosa; and both normal and remitted mucosa were defined as endoscopic healing (EH). MES 2 (marked erythema, absent vascular pattern, friability, erosions) and MES 3 (spontaneous bleeding, ulceration) were defined as active mucosae [10]. Endoscopic findings were judged by the score of the most inflamed mucosa in the colon. Endoscopic improvement was defined as a decrease of at least 1 in the MES score before and after tac treatment.

Histological inflammation was evaluated by one pathologist specializing in gastrointestinal pathology. The Nancy Histological Index was used to designate the degree of histological inflammation, with Grade 0 (no histologically significant disease), Grade 1 (chronic inflammatory infiltrate with no acute inflammatory infiltrate), Grade 2 (mildly active disease), Grade 3 (moderately active disease), and Grade 4 (severely active disease). Histological improvement was defined as a decrease of at least 1 in the Nancy Histological Index score before and after tac treatment [11].

Safety profile of therapeutic agents

The safety profile was evaluated based on all adverse events (AEs), including infections that occurred during all treatments. For this study, AEs attributable to thiopurines, which are often used to maintain remission, were also evaluated. Severe AEs (SAEs) were defined in this study as death, surgery because of an AE, an AE requiring admission to an intensive care unit or high care unit, and AEs that caused severe complications.

Study design

This retrospective study was conducted at a single institution in accordance with the ethical principles associated with the Declaration of Helsinki. It was approved by the Dokkyo Medical University Hospital Ethics Committee on July 18, 2023 (approval number: R-72-3J). The ethics committee of the Dokkyo Medical University Hospital deemed, because of the study's retrospective nature, that written informed consent was replaceable by the obligation of informing participants and giving participants the right to opt out. We provided participants with a means to opt out, instead of using informed consent, which safeguarded opportunities for research subjects to notify and publish research information related to our website. The option to opt out of the study was communicated to participants via our website with the following message: “Dokkyo Medical University Hospital is now conducting research using medical data from patients treated for ulcerative colitis. There will be no additional burden on patients for the conduct of this study. Additionally, we will conduct the research in compliance with laws and regulations related to the protection of patient privacy. If you do not want your medical data to be used for this study, please contact your doctor.”

The primary endpoint of the study was the analysis of factors contributing to successful induction of remission treatment with tac. The secondary endpoints were the CS-free remission rate at 52 weeks after tac induction and the endoscopic and histologic improvement rates after induction of remission.

Statistical analysis

Software (IBM SPSS Statistics 28®; IBM Japan Ltd.) was used for statistical analysis. Age, Lichtiger index, CRP, albumin, tac trough value, and time to reach therapeutic range are presented as mean ± SD. Pearson's χ-square test was used to compare gender, disease type, clinical course, endoscopic improvement, histological improvement, history of IM use, history of anti-TNFα biologics use, GMA combination rate, and 5-ASA intolerance. Fisher's exact test was used if the expected value was less than 5. The Mann-Whitney U test was used to compare age, clinical severity (Lichtiger index), CRP before treatment initiation, albumin before treatment initiation, the initial tac trough value, and the time to reach the therapeutic tac trough value. Significance was inferred for p-value < 0.05. A statistician evaluated the statistics calculated for this study.

## Results

Patient baseline characteristics

The background of the 67 patients is shown in Table 1. The mean age was 38.0 (12-70) years; 37 patients (55.2%) were male. Regarding the clinical course classification, 53 cases (79.1%) were relapsing-remitting type, 6 cases (9.0%) were chronic continuous type, and 8 cases (11.9%) were first attack type. The disease severity was severe in 17 cases (25.4%), moderate in 47 cases (70.2%), and mild in 3 cases (4.4%). The reasons for using tac were CS dependence in 25 cases (37.3%), CS resistance in 34 cases (50.8%), and CS-naïve in 8 cases (11.9%). A history of IM use was reported in 27 cases (40.3%), and a history of molecular-targeted agent use in 12 cases (17.9%). All 12 cases involved the use of anti-TNFα biologics.

**Table 1:** Background characteristics of the patients. IM: Immunomodulator; TNF: Tumor necrosis factor; GMA: Granulocyte and monocyte adsorption apheresis.

Characteristic (n=67)	
Age (years, mean ± SD)	38.0 ± 14.4
Sex (male)	37 (55.2%)
Extent of disease	
Proctitis type	0 (0%)
Left-sided type	17 (25.4%)
Pancolitis type	50 (74.6%)
Clinical course classification	
First attack type	8 (11.9%)
Relapse-remitting type	53 (79.1%)
Chronic continuous type	6 (9.0%)
Lichtiger index at the beginning of remission induction therapy	10.2 ± 2.6
Clinical severity at the beginning of remission induction therapy	
Mild	3 (4.4%)
Moderate	47 (70.2%)
Severe	17 (25.4%)
Corticosteroids	
Corticosteroids dependence	25 (37.3%)
Corticosteroids resistance	34 (50.8%)
Corticosteroids naive	8 (11.9%)
History of IM use	27 (40.3%)
History of molecular targeted agent (anti-TNFα antibody) use	12 (17.9%)
GMA combination rate	32 (47.8%)
5-aminosalicylic acid intolerance	7 (10.4%)

Remission induction rate with tac and factors contributing to remission induction

The remission induction rate at 8 weeks after tac administration was 67.2%. The remission induction rates at 8 weeks for the respective reasons for tac induction were 56.0% for CS dependence, 67.6% for CS resistance, and 100% for CS-naïve.

Analysis of factors contributing to successful induction of remission by tac showed that the Lichtiger index at the beginning of remission induction therapy was 9.5 ± 2.5 in the successful remission group and 11.5 ± 2.4 in the unsuccessful remission group (p = 0.002). The proportions of patients who had used IM were 28.9% in the successful remission group and 63.6% in the unsuccessful remission group (p = 0.006). The proportions of patients who had used anti-TNFα biologics were 8.9% in the successful remission group and 36.4% in the unsuccessful remission group (p = 0.006) (Table 2).

**Table 2:** Patient characteristics of the successful remission group and the unsuccessful remission group. CRP: C-reactive protein; Alb: Albumin; Tac: Tacrolimus; IM: Immunomodulator; TNF: Tumor necrosis factor.

Characteristic (n=67)	Successful remission group (n=45)	Unsuccessful remission group (n=22)
Age (years, mean ± SD)	37.0 ± 14.8	40.2 ± 13.3
Sex (male)	26 (57.8%)	11 (50.0%)
Extent of disease		
Proctitis type	0 (0%)	0 (0%)
Left-sided type	10 (22.2%)	7 (31.8%)
Pancolitis type	35 (77.8%)	15 (68.2%)
Clinical course classification		
First attack type	6 (13.3%)	2 (9.1%)
Relapse-remitting type	36 (80.0%)	17 (77.3%)
Chronic continuous type	3 (6.7%)	3 (13.6%)
Lichtiger index at the beginning of remission induction therapy	9.5 ± 2.5	11.5 ± 2.4
Clinical severity at the beginning of remission induction		
Mild	3 (6.7%)	0 (0%)
Moderate	33 (73.3%)	14 (63.6%)
Severe	9 (20.0%)	8 (36.4%)
CRP before treatment initiation (mg/dl, mean ± SD)	4.3 ± 4.9	3.7 ± 4.2
Alb before treatment initiation (g/dl, mean ± SD)	3.2 ± 0.7	3.0 ± 0.7
Initial Tac trough value (ng/dl, mean ± SD)	10.9 ± 6.7	9.9 ± 7.5
Duration to reach therapeutic range (days, mean ± SD)	5.2 ± 2.9	5.2 ± 2.5
History of IM use	13 (28.9%)	14 (63.6%)
History of molecular targeted agent (anti-TNFα biologics) use	4 (8.9%)	8 (36.4%)
5-aminosalicylic acid intolerance	3 (6.7%)	4 (18.2%)

Medium-term clinical response with tac

The CS-free remission rate at 52 weeks after tac administration was 61.2%. The CS-free remission rates at 52 weeks for the respective reasons for tac induction were 60.0% for CS dependence, 58.8% for CS resistance, and 75.0% for CS-naïve.

**Figure 2:**
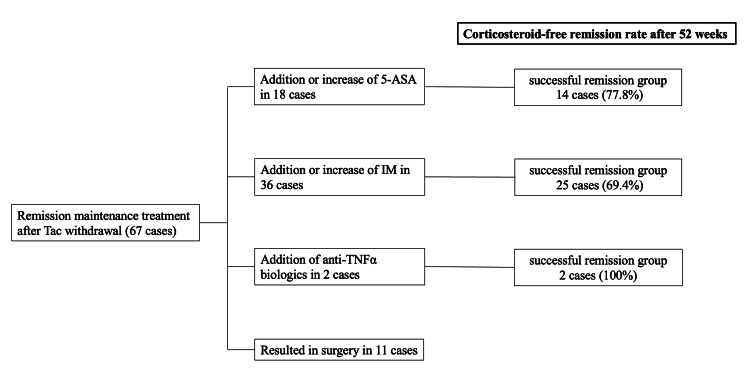
Corticosteroid-free remission rate after 52 weeks. Tac: Tacrolimus; 5-ASA: 5-aminosalicylic acid; IM: Immunomodulator; TNF: Tumor necrosis factor.

Remission maintenance treatment after tac withdrawal was intensified from the treatment before remission induction in all cases. This intensified maintenance included the addition or increase of 5-ASA in 14 cases, the addition or increase of IM in 25 cases, and the addition of anti-TNFα biologics in two cases. At 52 weeks, 14 cases (77.8%) of the 5-ASA addition/dose increase group, 25 cases (69.4%) of the IM addition/dose increase group, and 2 cases (100%) of the anti-TNFα biologics addition group had achieved CS-free remission (Figure 2).

Endoscopic and histologic evaluation before and after remission induction therapy with tac

Baseline characteristics of the 34 patients who were evaluable endoscopically and histologically before and within 8 weeks to 1 year after remission induction therapy with tac are presented in Table 3. Analysis of endoscopic improvement rates showed that 48.0% of patients in the successful remission induction group and 11.1% of patients in the unsuccessful remission induction group improved endoscopically, with a trend toward greater endoscopic improvement in the successful remission induction group (p = 0.051). The endoscopic improvement rates for the respective reasons to induce tac were 50.0% for CS dependence, 29.4% for CS resistance, and 40.0% for CS-naïve patients. Although 32.0% of the patients in the successful remission group and 22.2% of the patients in the unsuccessful remission group achieved histological improvement, no significant difference was found (p = 0.581). The histological improvement rates for the reasons for tac induction were 33.3% for CS dependence, 23.5% for CS resistance, and 40.0% for CS-naïve. Analysis of the achievement of CS-free remission after 52 weeks revealed a significantly higher rate of endoscopic improvement in the CS-free remission group (p = 0.037). However, no significant difference was found between the presence and absence of histologic improvement (p = 0.104).

**Table 3:** Baseline characteristics of patients evaluable endoscopically and histologically before and within eight weeks to one year after remission induction therapy. CRP: C-reactive protein; Alb: Albumin; Tac: Tacrolimus; IM: Immunomodulator; TNF: Tumor necrosis factor.

Characteristic (n=34)	Successful remission group (n=25)	Unsuccessful remission group (n=9)	P-value
Age (years, mean ± SD)	36.4 ± 14.7	34.7 ± 6.7	0.788
Sex (male)	14 (56.0%)	5 (55.6%)	0.982
Extent of disease			0.763
Proctitis type	0 (0%)	0 (0%)	
Left-sided type	7 (28.0%)	3 (33.3%)	
Pancolitis type	18 (72.0%)	6 (66.7%)	
Clinical course classification			0.544
First attack type	3 (12.0%)	0 (0%)	
Relapse-remitting type	20 (80.0%)	8 (88.9%)	
Chronic continuous type	2 (8.0%)	1 (11.1%)	
Lichtiger index at the beginning of remission induction therapy	9.7 ± 2.5	11.1 ± 3.0	0.151
Clinical severity at the beginning of remission induction			0.457
Mild	1 (4.0%)	0 (0%)	
Moderate	18 (72.0%)	5 (55.6%)	
Severe	6 (24.0%)	4 (44.4%)	
CRP before treatment initiation (mg/dl, mean ± SD)	4.7 ± 5.6	4.1 ± 4.1	0.645
Alb before treatment initiation (g/dl, mean ± SD)	3.2 ± 0.6	3.1 ± 0.5	0.489
Initial Tac trough value (ng/dl, mean ± SD)	10.6 ± 7.3	6.6 ± 3.3	0.216
Duration to reach therapeutic range (days, mean ± SD)	7.0 ± 2.7	5.7 ± 3.3	0.263
History of IM use	6 (24.0%)	7 (77.8%)	0.004
History of molecular targeted agent (anti-TNFα biologics) use	2 (8.0%)	2 (22.2%)	0.256
5-aminosalicylic acid intolerance	2 (8.0%)	3 (33.3%)	0.066
Endoscopic improvement rates	12 (48.0%)	1 (11.1%)	0.051
Histological improvement rates	8 (32.0%)	2 (22.2%)	0.581

Safety profile of therapeutic agents

The AEs related to the agents used for this study were as follows: tac was associated with one case of renal dysfunction and one case of hand numbness (3.0%); IM was associated with one case of pancreatitis and 1 case of mood disturbance (3.0%); 5-ASA was associated with seven cases of intolerance (10.4%). No adverse events were attributable to other agents. No SAEs were observed in this study.

## Discussion

This study demonstrated that the analysis of factors contributing to successful induction of remission with tac identified the non-use of IM and the non-use of anti-TNFα biologic agents as significant. In addition, analysis of the endoscopic improvement rate suggested that it may be a predictive marker for successful induction of remission and achievement of CS-free remission at 52 weeks.

The remission induction rate after eight weeks of tac was 67%. The results of this study are comparable to those described in earlier reports. For IBD treatment in Japan, tac was approved by the Japanese Ministry of Health, Labour and Welfare in 2009 as a remission induction agent for CS-resistant or dependent active UC, as well as for post-transplant immunosuppression and other treatments. The efficacy of tac for moderate to severe refractory UC has been verified through randomized trials. One report described a symptomatic improvement rate of 36% in the low-trough tac group (5-10 ng/mL) and 62% in the high-trough tac group (10-15 ng/mL) at two weeks of administration, compared to 10% in the placebo group [8]. Furthermore, Yamamoto S et al. showed that the group which achieved remission or clinical improvement within 30 days of tac had a significantly higher cumulative colectomy-free rate than the group which did not achieve improvement [12]. Using real-world data, Schmidt KJ et al. reported the efficacy of short-term tac treatment as 72% (94/130) in 130 patients with moderate to severe steroid-resistant UC in clinical remission after 12 weeks of treatment [13]. Based on worldwide reports, the short-term efficacy of tac for refractory UC is regarded as about 70%.

The primary endpoint of this study, the analysis of factors contributing to successful remission induction with tac, identified non-use of IM and anti-TNFα biologics. Takatsu N et al. reported that non-use of IM was a predictor of successful remission induction for 105 UC patients administered tac [14]. A patient with a history of IM and anti-TNFα biologics therapy might be expected to have high disease activity and might face difficulties in achieving remission induction. The European Crohn's and Colitis Organization guidelines state that a patient refractory to IM is unsuitable for rescue therapy with CyA, a calcineurin inhibitor similar to tac [15].

In this study, the CS-free remission rate after 52 weeks was 61.2%. The reason was that remission maintenance therapy was intensified after tac withdrawal, such as starting or increasing 5-ASA, starting or adding IM, and adding anti-TNFα biologics. The most suitable patients to consider for induction of remission with tac are those who have not used IM or molecular-targeted agents such as Janus kinase (JAK) inhibitors or anti-TNFα biologics preparations, leaving options open for remission maintenance therapy. One of the most remarkable features of this study was the detailed analysis of CS-free remission rates and how these remission rates could be optimized by additional treatment approaches. Pellet G et al. reported an interesting retrospective study of remission maintenance after remission induction with a calcineurin inhibitor [16]. According to their analysis, vedolizumab (VDZ) was used as a maintenance agent after induction remission with a calcineurin inhibitor in patients with CS-resistant UC, including many patients who had failed to induce remission with anti-TNFα antibodies. The analysis results showed a 68% colectomy-free rate at 12 months. Also, 44% of patients were able to continue maintenance therapy with VDZ. In addition, a 75% colectomy-free rate after 24 weeks in CS and anti-TNFα biologics resistant pediatric IBD patients was reported after using the combination of tac and VDZ [17]. The combination of molecularly targeted agents for maintenance therapy after remission induction with tac is expected to be beneficial.

Analysis of endoscopic improvement rates suggests that it might be a predictive marker for successful remission induction and achievement of CS-free remission at 52 weeks. No correlation was found between successful remission induction and CS-free remission at 52 weeks with histological improvement. This lack of correlation may be attributable to the absence of uniform criteria for tissue sampling sites. Endoscopic remission has been demonstrated to correlate strongly with colectomy avoidance rates and low relapse rates. Furthermore, endoscopic evaluation after therapeutic intervention is considered important for predicting subsequent prognoses [18-20]. Fukuda T et al. reported a lower relapse rate in the intervention group compared to the non-intervention group in patients with residual MES 1 despite clinical remission [21]. In recent years, endoscopic activity has shown a predominant correlation with fecal calprotectin and the fecal immunochemical test. These noninvasive biomarkers might serve as surrogate markers that can predict successful induction of tac remission and achievement of CS-free remission at 52 weeks in cases where there is hesitation against performing endoscopy [22].

There were some limitations to this study. First, the study was a retrospective study conducted at a single institution. Secondly, statistical analyses were constrained by the small number of cases. Future prospective multicenter studies on the prediction of long-term effects and endoscopic and histologic evaluation of tac should be conducted. Thirdly, the timing of endoscopic evaluations was not uniform. Coupled with varying maintenance therapies after tac withdrawal, this makes it difficult to compare endoscopic outcomes consistently across the study. The lack of standardized timing limits the ability to draw strong conclusions about the role of tac in promoting endoscopic remission in intermediate clinical responses.

## Conclusions

In conclusion, the analysis of factors contributing to successful induction of remission with tac identified the non-use of IM and anti-TNFα biologic agents as significant. Additionally, the analysis of the endoscopic improvement rate suggested that it may be a predictive marker for successful induction of remission and achievement of CS-free remission at 52 weeks.
